# DNA Aptamer Evolved by Cell-SELEX for Recognition of Prostate Cancer

**DOI:** 10.1371/journal.pone.0100243

**Published:** 2014-06-23

**Authors:** Yuanyuan Wang, Yun Luo, Tao Bing, Zheng Chen, Minhua Lu, Nan Zhang, Dihua Shangguan, Xin Gao

**Affiliations:** 1 Department of Urology, the third affiliated hospital of Sun Yat-sen University, Guangzhou, Guangdong, China; 2 Beijing National Laboratory for Molecular Sciences, Key Laboratory of Analytical Chemistry for Living Biosystems, Institute of Chemistry, Chinese Academy of Sciences, Beijing, China; CNR, Italy

## Abstract

Morbidity and mortality of prostate cancer (PCa) have increased in recent years worldwide. Currently existing methods for diagnosis and treatment do not make the situation improve, especially for hormone refractory prostate cancer (HRPC). The lack of molecular probes for PCa hindered the early diagnosis of metastasis and accurate staging for PCa. In this work, we have developed a new aptamer probe Wy-5a against PCa cell line PC-3 by cell-SELEX technique. Wy-5a shows high specificity to the target cells with dissociation constants in the nanomolar range, and does not recognize other tested PCa cell lines and other tested tumor cell lines. The staining of clinical tissue sections with fluorescent dye labeled Wy-5a shows that sections from high risk group with metastasis exhibited stronger fluorescence and sections from Benign Prostatic Hyperplasia (BPH) did not exhibit notable fluorescence, which suggests that aptamer Wy-5a may bind to protein related to the progression of PCa. The high affinity and specificity of Wy-5a makes this aptamer hold potential for application in diagnosis and target therapy of PCa.

## Introduction

Prostate cancer (PCa) is the most common non-cutaneous neoplasm in the male population worldwide [1]. Recently, several studies have shown that the incident of PCa is significantly higher than twenty years ago [2]. The update statistics shows that the morbidity of PCa has exceeded lung cancer and become the most common malignant tumor in men. More than 238,590 men will be diagnosed with PCa and 29,720 will die of metastatic PCa in the United States (US) in 2013, making it the second leading cause of cancer death in American men, behind lung cancer [3]. Unfortunately, most PCa patients in Asia had advanced local disease or metastases by the time they were diagnosed, and mortality rates of PCa may continue to rise in most Asian countries. This may be caused by the changes of life or dietary style and environment [4].

The performance of PCa in the early stage exhibits a relatively indolent cure in most patients [5]. This feature made PCa hard to be noticed and diagnosed in the early stage. When the patients are in pain, most of them have occured the bone metastasis. Initially, most PCa patients are sensitive to the androgen deprivation therapy (ADT), but the duration is heterogeneous, it could last from a few months to more than 3 years [6]. Eventually, PCa may evolve into Hormone Refractory Prostate Cancer (HRPC), which is resistant to conventional therapy. Successful cancer therapy is based on early diagnosis and accurate staging, both of which require suitable molecular probes and biomarkers [7]. Furthermore, the molecular level differences decide phenotype; personalized treatment is based on these different biomarkers to diagnose and distinguish exactly. But the lack of molecular probes for PCa cells hindered the early diagnosis of metastasis and accurate staging for PCa. Serum prostate specific antigen (PSA) is a biomarker for PCa, and has been widely used for the detection of PCa. The concentration of PSA is also associated with malignant degree and tumor recurrence. However, PSA is not a specific marker of PCa since its serum level increases with Benign Prostatic Hyperplasia (BPH) and is affected by many factors such as medication (Finasteride), urologic manipulations, inflammation, or even ejaculation [8], [9]. Although, along with the generalization of screening with prostate-specific–antigen (PSA) testing, the diagnostic rate and the early treatment was improved quickly, but the clinical trial in America and Europe show the screening has no effective on reducing mortality from PCa [10], [11]. So to improve clinical classification and personalized chemotherapy, it is still needs to find new biomarkers or probes with more specificity.

Recently, a new class of molecular probes termed aptamer has attracted much attention as molecular probes for disease diagnosis and therapy. Aptamers are single-stranded oligonucleotides that could fold into unique tertiary structures through various intramolecular interactions [12]. Similar to antibodies that are wildly used in clinic, aptamers can specifically bind to various targets that range from small organic molecules to proteins [13], [14]. Comparing to antibodies, aptamers have low-molecular weight, higher stability, rapid tissue penetration, lack of immunogenicity [15]–[18], and especially, aptamers can be chemically synthesized and modified [19]. These advantages make aptamers versatile tools for diagnostics [20], Image Cytometry [21] and targeted therapeutics [22].

Aptamers are generated by the SELEX technology (Systematic Evolution of Ligands by Exponential enrichment) [20], [23]. Cell-SELEX is an aptamer selection procedure using whole cells as target, which generates cell-specific aptamers by employing the differences at the molecular level between any two cell lines [19], [24]–[27]. By Cell-SELEX, a panel of aptamers that specically bind to target cell line can be identified without prior knowing the exact membrane proteins. In the process of enrichment, the living cells assure the targets existing on the membrane keep their nature formation, so the obtained aptamers will maintain the same affinity and specificity in their cellular applications [28]. In this paper, we describe the aptamer selection against PC-3 cells (a PCa cell line). Through cell-SELEX, we identified a DNA aptamer, which specifically binds to PC-3 cells and can distinguish BPH samples and high risk PCa samples from clinic.

## Results and Discussion

### Aptamer selection against PC-3 cells

**Figure 1:**
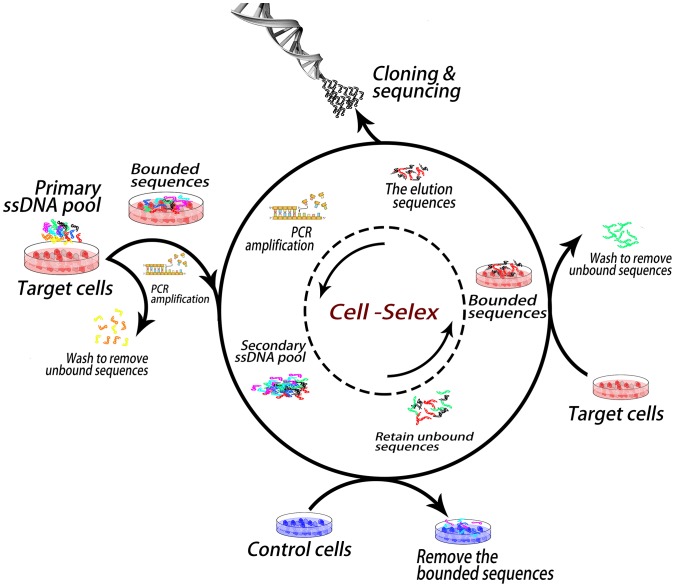
Schematic representation of the cell-based aptamer selection.

The process of aptamer selection is shown in Figure 1. The target cell line PC-3 is a typical cell line from androgen independent cancer patient with osseous metastasis. Androgen independent cancer patients with osseous metastasis are the most difficult to treat. In order to generate aptamers with high specificity, we used more than one kind of cell line as negative control for counter selection, including nontumor-immortalized prostate epithelial cells line RWPE-1, human hepatic carcinoma cell line SMMC-7721 and Human cervical cancer cell line Hela.

**Figure 2:**
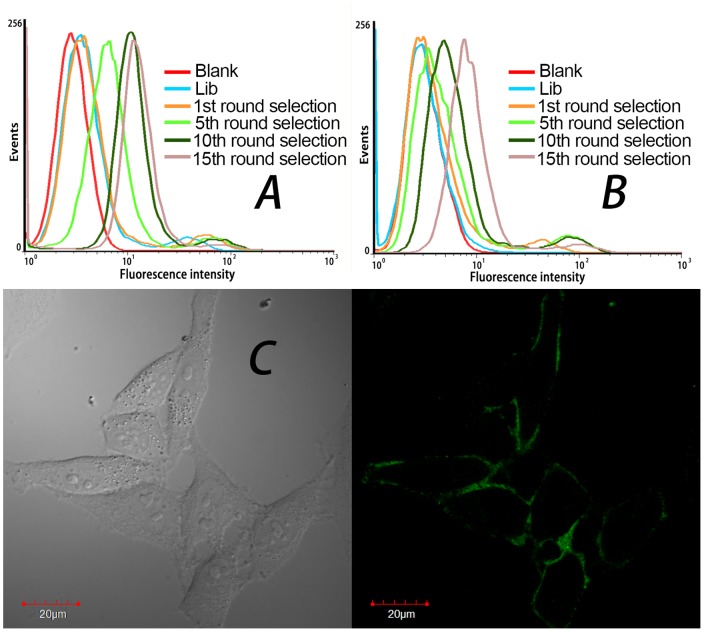
Binding assay of selected pools to PC-3 and SMMC-7721 cells. Flow cytometry assay of selected pools binding to target cell line PC-3 (A) and negative control cell line SMMC-7721(B), Blank is the background fluorescence of untreated cells. Lib is FITC-labeled DNA library as negative control. (C) Confocal imaging of PC-3 cells stained by the 15th-round selected pool labeled by FITC (×100 oil immersion lens).

For the first round selection, the target cells were incubated with the random DNA library pool. After washing, the remained sequences on cells were amplified by polymerase chain reaction (PCR), and separated into single-stranded sequences for the second round selection. From the second round selection, the negative cells were incubated with the enriched pool before the target cell binding in order to eliminate the sequences that bound to the common molecules present on the surface of target cells and control cells. For every two or three rounds of selection, the aptamer enrichment was monitored by flow cytometry and confocal imaging. If aptamer sequences were enriched, the fluorescence intensity on the surface of PC-3 cells became stronger after incubation of cells with the selected pools. As shown in Figure 2A, the fluorescence intensity on the surface of PC-3 cells greatly increased in the first ten rounds of selection, and the fluorescence enhancement on the control cells (SMMC-7721, Figure 2B) was much smaller, indicating that aptamers for target cells were greatly enriched. However, after performed further five rounds of selection, although the fluorescence on PC-3 cells was still stronger than on control cells, the fluorescence enhancement on PC-3 cells became slower and that on control cells became faster, which suggests that more nonspecific sequences that bound to both cell lines were enriched. Confocal imaging (Figure 2C) showed that aptamers bound on the membrane of the target cells. After another two rounds of selection with stronger counter selection, the 17th round pool was cloned and 50 clones were sequenced.

### Identification of aptamers against target cells

**Figure 3:**
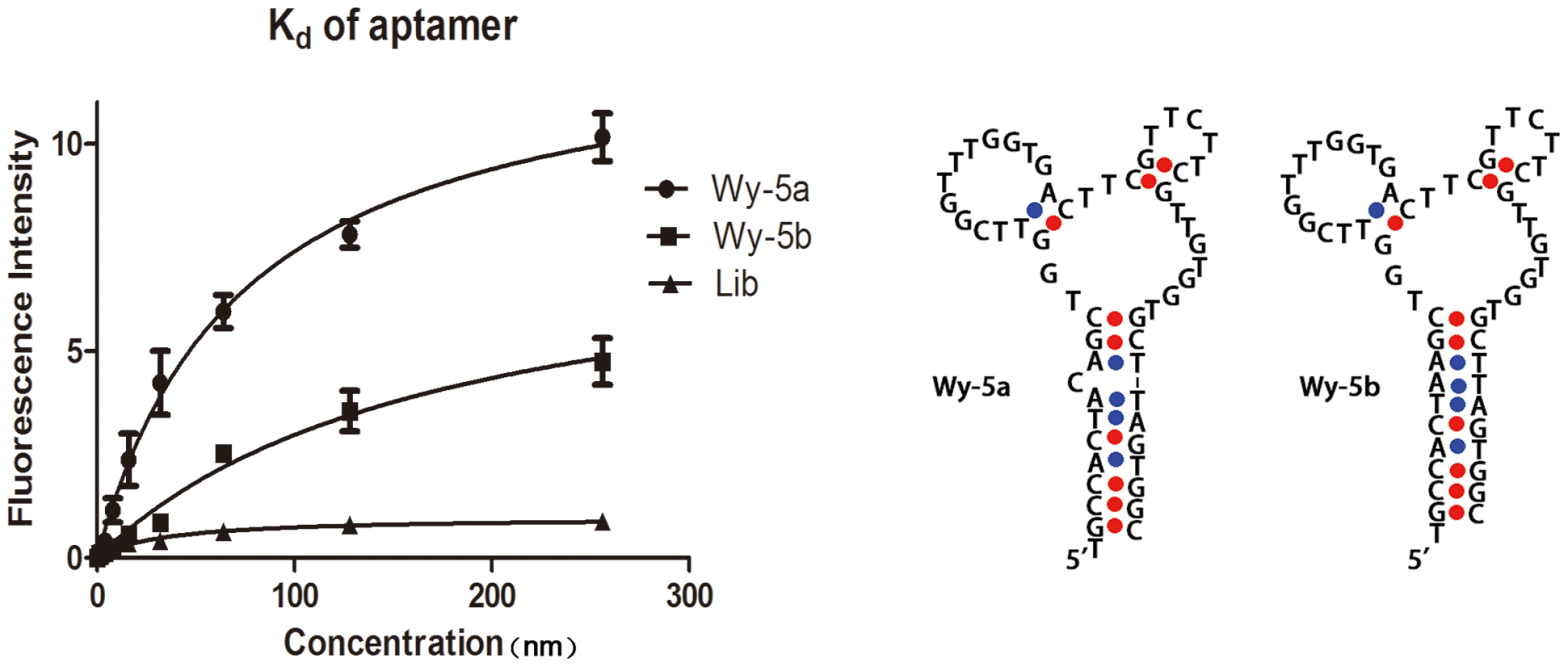
Binding curves of FITC-labeled aptamer sequences to PC-3 cells (Wy-5a and wy-5b, left) and the predicted secondary structure of Wy-5a and wy-5b (right). The concentrations of DNA are 0, 1.0, 2.0, 4.0, 8.0, 16, 32, 64, 128 and 256-labeled DNA library (Lib). The predicted secondary structure of Wy-5a and wy-5b (right), were rebuild by the tool provided by Integrated DNA Technologies (IDT). [www.IDTdna.com/page/scitools].

After alignment, the obtained sequences of 50 clones were found to distribute into 5 families according to the similarities of thier sequences. Through analyzing the predicted secondary structure of the sequences in each family [29]–[31], six sequences were truncated and synthesized for binding assay (sequences not shown). Flow cytometry assay showed that a sequence, Wy-5a exhibited the strongest binding to PC-3 cells and the weakest binding to other cell types (Figure S1); therefore this sequence was further characterized. The secondary structure prediction [32] showed that Wy-5a adopted a stem-loop structure with a one-base bulge on the stem (Figure 3). Removing the one-base bulge, a perfect stem-loop structure sequence Wy-5b (Table 1) was also synthesized for further characterization. Binding assay showed that Wy-5a and Wy-5b specifically bound to PC-3 cells (Figure 3). The equilibrium dissociation constants (*K*_d_) of Wy-5a and Wy-5b were calculated to be 73.59±11.01 and 173.1±44.67, indicating that aptamer Wy-5a has higher affinity to PC-3 cells than Wy-5b. The higher affinity of Wy-5a suggests that the one-base bulge on the stem of Wy-5a may involve the binding to its target. Because of the higher affinity of Wy-5a, it was further characterized as a novel probe for PCa detection.

**Table 1:** Sequences and *K*_d_s of selected aptamers for the PC-3 cells.

Aptamers	Sequences	*K*_d_ (nM)
**Wy-5a**	TGCCACTACAGCTGGTTCGGTTTGGTGACTTCGTTCTTCGTTGTGGTGCTTAGTGGC	73.59±11.01
**Wy-5b**	TGCCACTAAGCTGGTTCGGTTTGGTGACTTCGTTCTTCGTTGTGGTGCTTAGTGGC	173.1±44.67

### Specificity of Selected Aptamers

**Figure 4:**
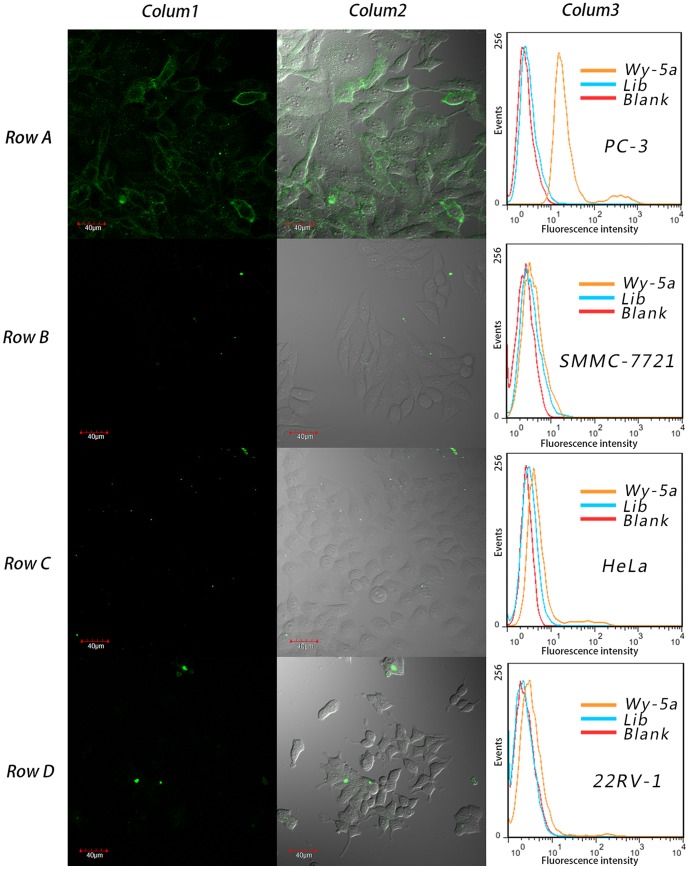
The specificity assay of aptmer Wy-5a to different cell lines. Row A, target cell line PC-3; Row B, SMMC-7721; Row C, HeLa; Row D, 22RV-1. Colum 1, fluorescence images; Colum 2, overlay of fluorescence images and bright field images, Colum 3, Histogram Plot of Flow cytometry. Blank is the background fluorescence of untreated cells respectively; Lib is FITC-labeled DNA library as negative control.

The specificity of Wy-5a to other tumor cell lines was investigated by flow cytometry and confocal imaging (Figure 4). Among all the tested cell lines, Wy-5a only bound to PC-3 cell line, did not bind to other PCa cell lines (such as DU145, from PCa brain metastasis with moderate metastatic potential; 22RV-1, from local PCa without metastatic potential),and other cancer cell lines, including Lung cancer cell line (A549), Human breast cancer cell line (MCF-7), Human cervical cancer cell line (HeLa), Hepatoma cell line (SMMC-7721), Colon cancer cell lines (LoVo and HCT-8), leukemia cell line (Jurkat) and chronic myelogenous leukemia cell line (K562) (table S1). These results indicate that aptamer Wy-5a has excellent specificity to the target cell line. The high specificity makes Wy-5a has the potential for application in detection of metastasis PCa or a tool for target therapy.

### The binding sites of the aptamer on the target cells

**Figure 5:**
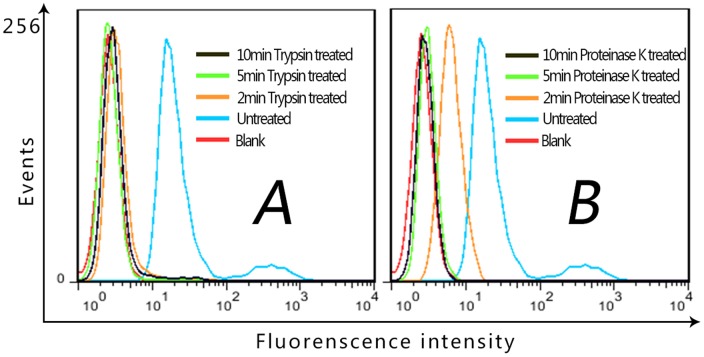
The binding of selected aptamer to PC-3 cells pre-treated with trypsin (A) or proteinase-K (B). After treatment with trypsin or proteinase K, PC-3 cells almost did not bind Wy-5a. Blank is the background fluorescence of untreated cells respectively.

The confocal imaging has shown that the obtained aptamers bound on cell surface (Figure 2C and Figure 4 Row A), thus a proteinase digestion experiment was carried out to verify whether the target molecule is a membrane protein. As shown in Figure 5, after treatment with trypsin or proteinase K for 2 min, PC-3 cells almost did not bind Wy-5a, indicating that the target molecule of aptamer Wy-5 is a membrane protein.

### The internalization of Wy-5a

**Figure 6:**
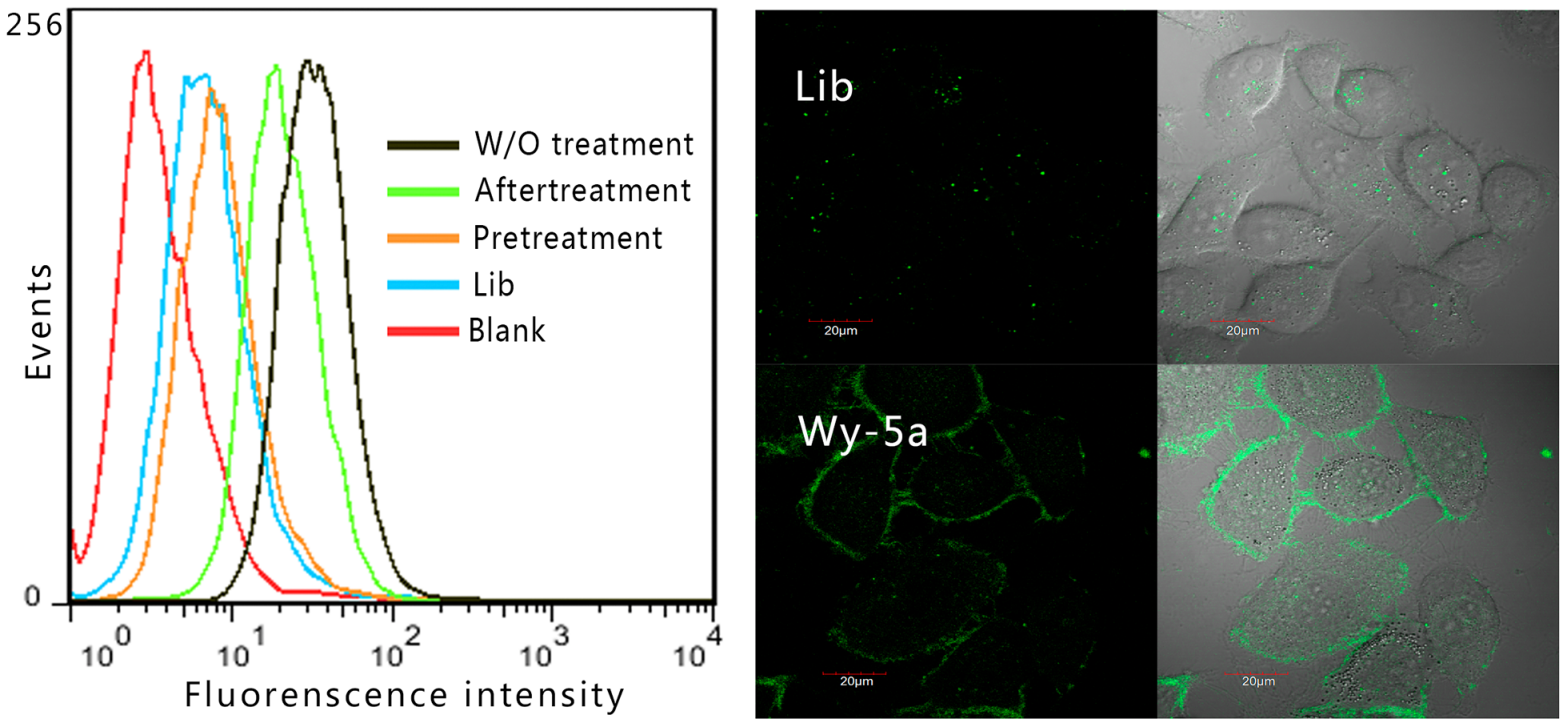
Cell-specific internalization of aptamer Wy-5a assessed by flow cytometry assay (left) and confocal imaging (right). W/O treatment: Cells were incubated with Wy-5a; Pretreatment: cells were pretreated by trypsin and then incubated Wy-5a; aftertreatment: cells were incubated Wy-5a and then treated by trypsin; Lib: negtive control, cells were incubated with FITC-labeled DNA library; blank: is the background fluorescence of untreated cells.

Previous studies have shown that oligonucleotides longer than 25 bases could not freely penetrate the membrane [33], [34]. Wy-5a is a 52-base oligonucleotide, it targets a membrane protein. In order to investigate whether Wy-5a can internalize into cells through receptor-mediated endocytosis, PC-3 cells were incubated with Wy-5a at 37°C for 2 h. The increase of tempreture from 4°C to 37°C did not much affect the binding of Wy-5a to PC-3 cells (Figure S2). The flow cytometry assay (Figure 6) showed that pre-treatment of cells with trypsin caused almost complete loss of aptamer binding (compared with unbound DNA labrary). However, treatment of cell with trypsin after aptamer binding only caused partial loss of aptamer binding, suggesting that some Wy-5a might internalize into cells. In the confocal image of PC-3 cell after incubation with Wy-5a at 37°C for 2 h (Figure 6), strong fluorescence signal was observed on cell membrane and weak fluorescence signal was found in whole cells. But the the confocal image of cells icubated DNA library did not show significant fluorescence signal except for some nonspecific bright spots dispersedly adsorbed on cells. These results suggest that Wy-5a may be involved in receptor-mediated endocytosis.

### The staining of clinical PCa slides

**Figure 7:**
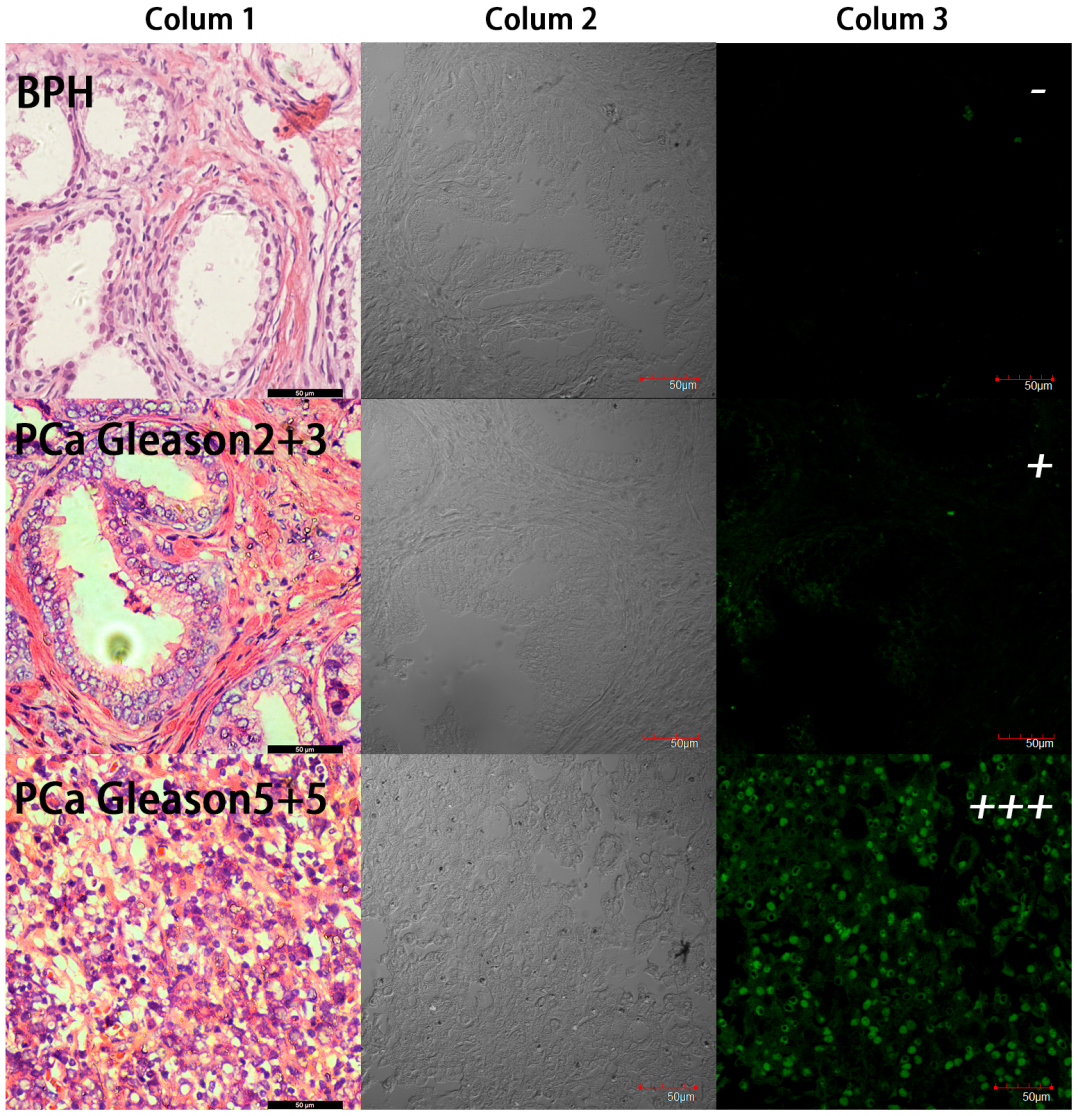
The clinical slides stained by 250-labeled Wy-5a. Rarely fluorescence signal (−) was observed on BPH slides. In well-differentiated prostate cancer, Gleason score: 2+3, no proof of other organ metastasis, very weak fluorescence signal (+) was observed. In low-differentiated prostate cancer patient, Gleason score: 5+5, with bone metastasis, strong fluorescence signal (+++) was observed. (Colum 1) HE staining of paraffin section from clinical sample, (Colum 2) Bright field of confocal, (Colum 3) Fluorescence signal of confocal.

Above results hav shown that aptamer Wy-5a has excellent specificity to PC-3 cells over DU145 and 22RV-1 cells. PC-3 cell line was initiated from a bone metastasis of a grade IV androgen independent PCa patient. Furthermore, the skeletal involvement is the most common metastatic site in advanced-stage PCa, observed in up to 80% of patients [35]. DU145 was derived from PCa brain metastasis with moderate metastatic potential, and are not hormone-sensitive [36]; 22RV-1 is a human PCa cell line without metastatic potential derived from a xenograft in mice [37]. Therefore the target protein of Wy-5a may have correlation with aggressiveness or metastasis. Thus aptamer Wy-5a may have the potential in tumor classification and staging. In order to test the feasibility of Wy-5a for tumor classification and staging, we used this aptamer to stain PCa tissue sections from clinical patients. The negative control is BPH tissues, a prevalent noncancerous change in aged man. Tumor tissues were gotten from patients with PCa (identified by senior pathologist, the third affiliated hospital of Sun Yat-Sen University). The sections were cut from formalin-fixed paraffin-embedded (FFPE) tissues, and the membrane antigens were repaired by boiling EDTA Antigen Retrieval Solution before staining. As shown in Figure 7, after stained by FITC (fluorescein isothiocyanate) labeled aptamer Wy-5a, the cancer tissues exhibited strong fluorescence signal. However the fluorescence signal was hardly observed on the slide of BPH tissue.

The staining of all slides was summarized in Table 2. Totally, all the 10 BPH slides from different patients showed negative results and 20 cancer slides out of 28 cases of cancer tissue showed positive results. Only 8 cases of cancer slides could not be stained by the aptamer. Comparing the Gleason score of cancer tissues and the medical record of the patients, we found an interesting phenomenon: the slides from patients with high Gleason score (>6) with bone metastasis showed stronger fluorescence on the cell membrane than low score (≤6) without metastasis (Figure 7). This indicated that the target protein of Wy-5a highly expressed in these high score patients with metastasis. The Gleason grading system is a powerful tool to prognosticate and aid in the treatment of men with PCa. Based on pathological structure of gland epithelial, the tissue image is classified into five types. From the first to the fifth type, the degree of differentiation gradually reduced [38]. Since Gleason score reflects the malignancy degree of PCa, these results may imply that the target protein of Wy-5a maybe have some relationship with the malignant degree or aggressiveness or progression of tumor. Further protein identification and evaluation will be carried out to illuminate this issue. This set of results suggests aptamer Wy-5a has the potential to serve as probe for the diagnosis of PCa.

**Table 2:** Assessment of clinical slides stained by dye labeled aptamer.

	BPH benign lesion (10 cases)	Low Gleason score* without metastasis (13 cases)	High Gleason score** with metastasis (15 cases)
−	10	5	3
+	—	6	1
++	—	2	5
+++	—	—	6

*Low Gleason score: ≤6, **high Gleason score>6.

‘−’ no staining; ‘+’ faint incomplete staining; ‘++’ moderate complete membrane staining;

‘+++’ strong complete membrane staining [45].

The PCa patients with recurrence or in advanced stage will eventually become HRPC and show no responds to ADT [6]. The development of its drug resistance in addition to hormone refractoriness, make the effect of treatment frustrating [39]. However, heterogeneous duration indicated that the types and manifestations of the patients are various. Accurate classification of type or grade is helpful to improve the efficiency of therapy. Therefore more diagnostic reagents are needed in order to choose the optimum personalized therapy. Some chemotherapeutics are effective in vitro, but lack of cell-selectivity, serious toxicity and side effects limited their usage in vivo. Aptamer mediated targeted medicine may be a good solution. Until now days, only one RNA aptamer aiming at PCa has been generated by traditional SELEX process, i.e. aptamer against PSMA (Prostate Specific Membrane Antigen, only expressed in LNCaP cell line) [40]. For the typically cell line PC-3, there is still no oligonucleotide aptamer evolved. Recently aptamer based drug delivery has shown good results [41]. The high affinity and specificity to PC-3 cell line and to cancer tissues with high risk and metastasis imply that DNA aptamer Wy-5a hold potential in the field of classification, detection and target therapy of PCa patients.

## Experimental Section

### Cell culture

Twelve established cell lines were used: RWPE-1 is an immortalized normal Human prostate epithelial cell line. PC-3, DU-145 and 22RV-1 are human PCa cell lines. SMMC-7721 is a human hepatic carcinoma cell line. LoVo and Hct-8 cells are colorectal carcinoma cell lines. HeLa is a Human cervical cancer cell lines. A549 is a Human lung cancer cell line. MCF-7 is a Human breast cancer cell line, Jurkat is an acute T cell leukemia cell line, K562 is a chronic myelogenous leukemia cell line. RWPE-1, PC-3, DU-145, 22RV-1, SMMC-7721 and LoVo cell lines were purchased from Typical culture preservation commission cell bank, Chinese academy of sciences (Shanghai, China). HeLa, A549, MCF-7, Jurkat and K562 were purchased from the Institute of Basic Medical Science at the Chinese Academy of Medical Sciences (Beijing, China). RWPE-1 was cultured in Keratinocyte Serum Free Medium (K-SFM) with bovine pituitary extract (BPE) and human recombinant epidermal growth factor (EGF), the other tumor cell lines were cultured in RPMI 1640 culture medium supplemented with 10% fetal bovine serum (FBS, GIBCO) and 100 units/mL penicillin streptomycin (Sigma). All cell lines were maintained at 37°C and 5% CO_2_ incubator. In the whole process, every important index of growing on cells must keep stable and cells were kept in a good state, which establish a stable basis for further experiments.

### Buffer

Washing buffer was prepared by adding 5 mmol MgCl_2_ and 4.5 g glucose into 1 L 0.01 M PBS (contain 8 g NaCl, 0.2 g KCl, 3.58 g Na_2_HPO_4_·12H_2_O, 0.272 g KH_2_PO_4_ per 1 L ddH_2_O. pH = 7.4) without calcium and magnesium. The binding buffer was prepared by adding 1 mg/mL bovine serum albumin (BSA, Sigma) and 0.1 mg/mL Herring Sperm DNA (Invitrogen) to washing buffer.

### SELEX ssDNA library and PCR primers

The HPLC-purified library contained a central randomized sequence of 45 nucleotides (nt) flanked by 20-nt primer hybridization sites (5′-ACGCTCGGATGCCACTACAG-45nt-CTCATGGACGTGCTGGTGAC -3′). A fluorescein isothiocyanate (FITC)-labeled 5′-primer (5′-FITC-ACGCTCGGATGCCACTACAG-3′) and a biotinylated (Bio) 3′-primer (5′-Bio-GTCACCAGCACGTCCATGAG-3′) were used in the PCR reactions for the synthesis of double-labeled, double-stranded DNA molecules. After denaturing in alkaline conditions (0.2 M NaOH), the FITC- conjugated sense ssDNA aptamer is separated from the biotinylated antisense ssDNA strand by streptavidin-coated sepharose beads (Streptavidin Sepharose High Performance)(GE Healthcare, Sweden) and used for next round selection or binding assay. The selection process was monitored using flow cytometry assay and confocal imaging.

### Procedure of SELEX

The process of cell-SELEX is similar to that described previously [42]. In briefly, the process was carried out as follow steps (Figure 1). Before each round of SELEX, the target cells PC-3 were cultured to 80∼90% confluence and the negative cells were cultured to complete confluence in 60 mm petri dish and washed three times with precooled PBS. 10 nmol initial ssDNA library dissolved in 1 mL of binding buffer was denatured at 95°C for 5 min and kept in ice bath for 15 min, then placed in room temperature about half an hour before used. After incubating the library pool with the target cells at 4°C for 1 h, the initial library solution was removed. After washed with washing buffer the adhesive cells were scraped off and collected in a 2 mL tube, washed again. The bounded DNAs were eluted by adding 500 µl of ddH_2_O into the tube, and heating to 95°C for 5 min. After centrifugation, the supernatant was used as the template and amplified by the PCR with the primers labeled by FITC or biotin (10–15 cycles of 30 sec denature at 95°C, 30 sec annealing at 59°C, and 30 sec extension at 72°C, the last round followed by 5 min at 72°C; keep the production at 4°C. The Taq polymerase and dNTPs were obtained from Takara). The FITC-labelled sense ssDNA pool was separated from the PCR product by streptavidin-coated sepharose beads for the next round selection. From the second round of selection, the negative cells were incubated with the ssDNA pool first for counter selection, then the negative cells bound with unspecific sequences were removed by centrifuge. The supernatant was incubated with target cells to enrich the specific sequences. The selection process was monitored using flow cytometry and confocal imaging. From the 2nd to 5th round, RWPE-1 cells were used for counter selection; from the 6th to 8th round, SMMC-7721 were used for counter selection; from the 9th to 10th round selection, Hela cells were used for counter selection. After 10th round, HeLa and SMMC-7721 cells were used for counter selection separately in each round. To obtain aptamers with high affinity and specificity, the pressure of selection was enhanced gradually from 1st to 15th selection by decreasing the amount of the ssDNA pool (from 100 pmol to 50 pmol), the incubation time for the target cells (from 60 min to 30 min), and the target cell number (from 7.5 million/10 cm dish to 1 million/3.5 cm dish) and by increasing the number of washes (from two to three). In the last two rounds of strengthened selection (16th–17th), we reduced the pool to 30 pmol, incubation time to 15 min and increase the washing to four times with stronger shaking. Totally 17 rounds of selection were performed before sequencing. The clone and sequencing were performed by Sangon Biotechnology Co., Ltd.,

### Flow cytometric analysis

To monitor the enrichment of aptamer along with the progress of SELEX, the target cells and negative cells was cultured into monolayers with 80∼90% confluence, then dissociated the cells by 0.02% EDTA after washed by PBS once at room temperature. After washed with cold washing buffer twice, cells (2×10^5^) were incubated with FITC-labeled ssDNA pools or selected DNA sequences (0.25 µM) in 200 µL of binding buffer at 4°C for 30 minutes. Before flow cytometry assay, the cells were washed twice by cold washing buffer and filtered with a 400 mesh cell sieve. The fluorescence was determined with FACScan cytometer (Becton Dickinson) by counting 1×10^4^ events. The FITC labeled library pool was used as a negative control.

### Staining of human tumor tissue sections using selected aptamer

Paraffin tissue blocks were provided by the pathology department of 3rd affiliated hospital, Sun Yat-sen university (Guangzhou China). The pretreatment of the sections (5 µm thick) was performed as described previously [43], [44]. The antigen retrieval process is similar with Immunohisto-chemistry. The tissue sections were deparaffinated in xylene twice (10 min each time) after kept in constant temperature oven at 60°C for 20 min. Use the gradient ethanol (100%, 95% and 70%) to rehydrate the section and rinse the slides in deionized water. After wash in PBS for 5 min, all the sections were put in TE buffer (contain 10 mmol Tris, 1 mmol EDTA per 1 L ddH_2_O, pH = 8.0) and quickly heated to boiling by microwave oven, then keep the temperature at 95°C for 15 min to retrieve antigens and natural cooling to room temperature, then washed three times with fresh PBS. After histological process, The tissue sections were incubated with binding buffer containing 20% FBS and 0.1 mg/mL Herring Sperm DNA at room temperature for 60 min and then incubated with 250 nM FITC-labeled aptamers in binding buffer 60 min at 4°C. Then these tissue sections were washed three times with fresh PBS, dehydrated and sealed by Antifade Polyvinylpyrrolidone Mounting Medium. Finally, the stained sections were imaged by spinning-disk confocal fluorescence microscopy (Olympus IX71, Japan). FITC was excited with a 488 nm argon ion laser (Mells Griot, CA).The fluorescence signals were observed by a 40× objective (NA 0.90, UPLSAPO, Olympus, Japan). The images were analyzed by FV10-ASW Version 3.1.

### Affinity and specificity of the aptamer

14 cell lines were used to test the specificity of the aptamers by flow cytometry assay as described above. In order to test the affinity of different aptamers to PC-3cell, different concentration of aptamers were incubated with 2×10^5^ cells at 4°C, and then analyzed by flow cytometry. The mean fluorescence intensity of each sample was subtracted the mean fluorescence intensity of background. The equilibrium dissociation constants (*K*_d_) of the aptamer–cell interaction were obtained by fitting the dependence of fluorescence intensity of specific binding on the concentration of the aptamers to the equation *Y* = *B* max *X*/(*K*_d_+*X*), using SigmaPlot (Jandel, San Rafael, CA) [25].

### Proteinase treatment for cells

PC-3 cells were washed twice on dish by PBS at room temperature, dissociated by 0.02% EDTA. After washed, 2×10^5^cells incubated with 200 µl of 0.05% trypsin or 0.1 mg/ml proteinase K at 37°C for 2, 5 and 10 min respectively. The reaction was terminated by adding complete medium. After wash, the treated cells were incubated with aptamer and applied for flow cytometry assay as described previously.

### Confocal image of cells

The confocal fluorescence microscopy was used to observe the binding of aptamers to living cells. 2×10^4^ cells were cultured in 35 mm Glass Bottom Dish for more than 1 day to get a well extension. After washed by cold washing buffer, the cells were incubated with aptamers (0.25 µM) at 4°C for 30 min. After washed twice, cells were imaged by spinning-disk confocal fluorescence microscopy (Olympus IX71, Japan). FITC was excited with a 488 nm argon ion laser (Mells Griot, CA).The fluorescence signals were observed by a 40× objective (NA 0.90, UPLSAPO, Olympus, Japan). The images were analyzed by FV10-ASW Version 3.1.

### The internalization of the aptamer

The target cell PC-3 was dissociated by 0.02% EDTA. After washing, cells were divided into 5 tubes. One tube without any treatment as blank; one tube was incubated with 0.25 µM FITC-labeled DNA Library as negtive control; one tube was incubated with 0.25 µM FITC-labeled Wy-5a as positive control; one tube was pre-treated by 0.05% Trypsin for 10 min and then incubated with 0.25 µM FITC-labeled Wy-5a (pre-treatment); one tube was incubated with 0.25 µM FITC-labeled Wy-5a and then treated by 0.05% Trypsin for 10 min (after-treatment). All the incubations with DNA were performed at 37°C for 2 h in serum-free RPMI 1640 medium. Then the five samples were applied for flow cytometry assay as described above. For the confocal observation, PC-3 cells were incubated with 0.25 µM FITC-labeled Lib or Wy-5a in serum-free RPMI 1640 medium at 37°C for 2 h then applied for imaging as described above.

## Supporting Information

Figure S1 **Flow cytometry assays of Cells after incubation with tdifferent aptamers.** Blank: is the background fluorescence of untreated cells.
(TIF)

Figure S2 **Flow cytometry assay of PC-3 cells after incubation with Wy-5a at 4°C or 37°C.** The binding ability of Wy-5a show no difference at 4°C or 37°C. Blank: is the background fluorescence of untreated cells.
(TIF)

Table S1 **Summary of Wy-5a binding to different cell lines.**
(DOC)
