# Supplementary figures and images for: DNA Aptamer Evolved by Cell-SELEX for Recognition of Prostate Cancer
Source: PLoS One. 2014 Jun 23;9(6):e100243. doi: 10.1371/journal.pone.0100243 (PMC4067300; doi:10.1371/journal.pone.0100243)

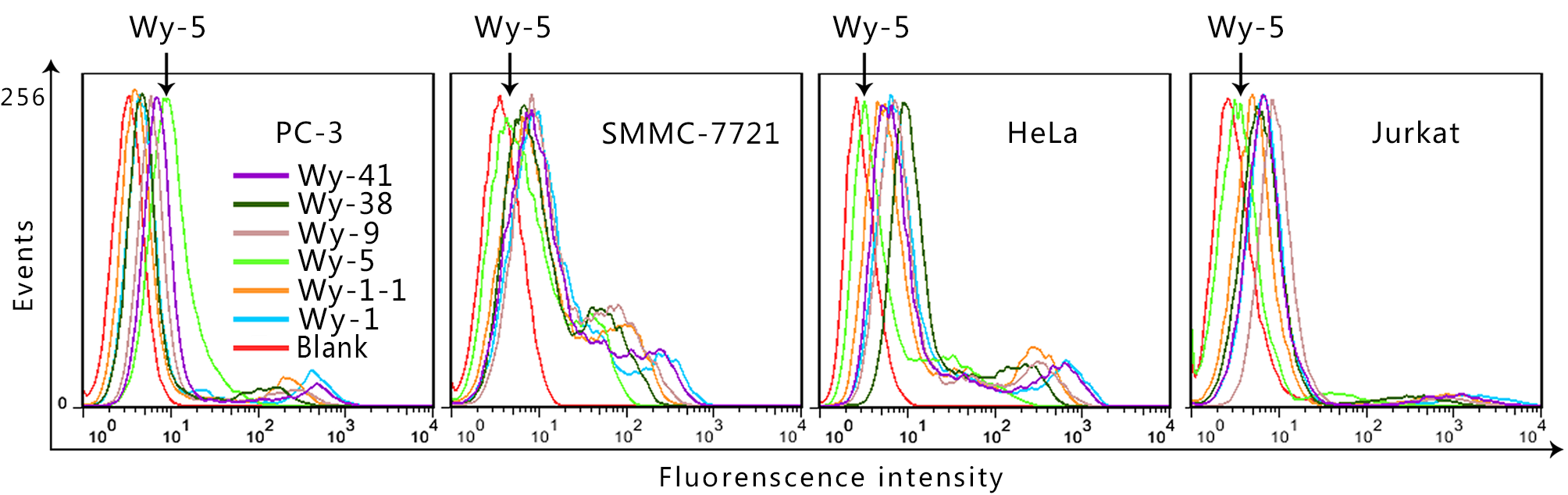

Supplement: Figure S1 — Flow cytometry assays of Cells after incubation with tdifferent aptamers. Blank: is the background fluorescence of untreated cells. (TIF) [file pone.0100243.s001.tif]

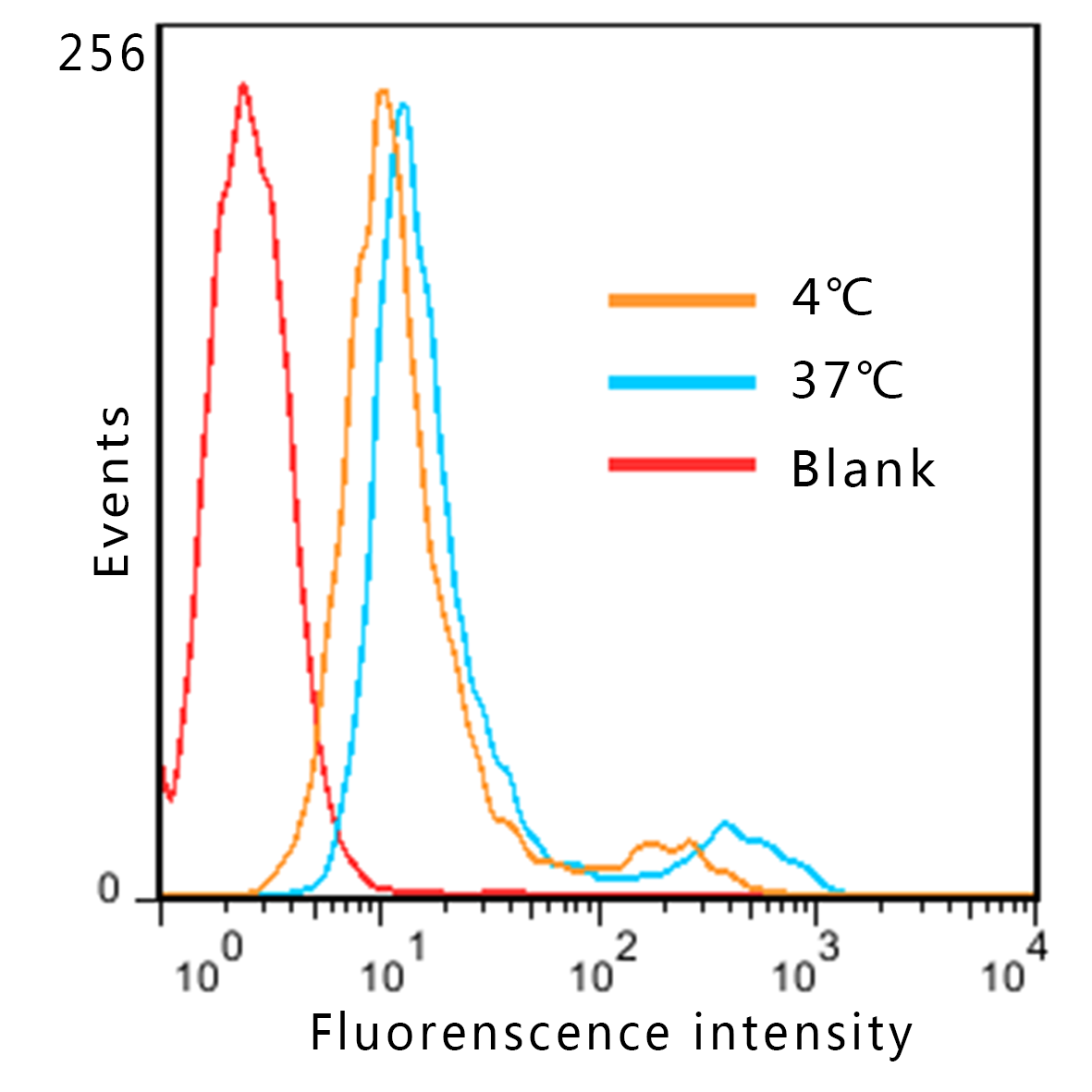

Supplement: Figure S2 — Flow cytometry assay of PC-3 cells after incubation with Wy-5a at 4°C or 37°C. The binding ability of Wy-5a show no difference at 4°C or 37°C. Blank: is the background fluorescence of untreated cells. (TIF) [file pone.0100243.s002.tif]
