# Supplementary material for: DNA Aptamer Evolved by Cell-SELEX for Recognition of Prostate Cancer
Source: PLoS One. 2014 Jun 23;9(6):e100243. doi: 10.1371/journal.pone.0100243 (PMC4067300; doi:10.1371/journal.pone.0100243)
Supplement: Table S1 — Summary of Wy-5a binding to different cell lines. (DOC) [file pone.0100243.s003.doc]

**Supporting Data:**

Table S1. **Summary of Wy-5a binding to different cell lines**

| Cell line | Aptamer binding |
| --- | --- |
| PC-3 | **++++** |
| DU-145 | **-** |
| 22RV-1 | **-** |
| SMMC-7721 | **-** |
| HeLa | **-** |
| LoVo | **-** |
| HCT-8 | **-** |
| MCF-7 | **-** |
| A549 | **-** |
| Jurkat | **-** |
| K562 | **-** |

In the flow cytometry analysis, a threshold of fluorescence intensity was chosen so that 99% of cells interacted with unselected DNA library would have fluorescence intensity below it. When the aptamer was interact with cells, the percentage of the cells with fluorescence above the set threshold was used to evaluate the binding capacity of the aptamer to the cells. -, <10%; **+**, 10–35%; **++**, 35–60%; **+++**, 60–85%; **++++**, >85%.
